# A WWP2–PTEN–KLF5 signaling axis regulates odontoblast differentiation and dentinogenesis in mice

**DOI:** 10.1016/j.jbc.2022.102220

**Published:** 2022-07-01

**Authors:** Jing Fu, Xiaobo Zhang, Huiwen Zheng, Guobin Yang, Zhi Chen, Guohua Yuan

**Affiliations:** 1 The State Key Laboratory Breeding Base of Basic Science of Stomatology and Key Laboratory for Oral Biomedicine of Ministry of Education, School and Hospital of Stomatology, Wuhan University, Wuhan, China; 2 Frontier Science Center for Immunology and Metabolism, Wuhan University, Wuhan, China

**Keywords:** odontoblasts, differentiation, dentin, tooth development, ubiquitination, phosphatase and tensin homolog, Krüppel-like factor 5, WW domain-containing E3 Ubiquitin-protein ligase 2, 2W, 2 weeks, ALP, alkaline phosphatase, ARS, alizarin red S, co-IP, coimmunoprecipitation, IF, immunofluorescence, IHC, immunohistochemistry, KLF5, Kruppel-like factor 5, mDPC, mouse dental papilla cell, PLA, proximity ligation assay, PN, postnatal, PTEN, phosphatase and tensin homolog, WWP2, WW domain–containing E3 Ubiquitin-protein ligase 2

## Abstract

WW domain–containing E3 Ubiquitin-protein ligase 2 (WWP2) has been found to positively regulate odontoblastic differentiation by monoubiquitinating the transcription factor Kruppel-like factor 5 (KLF5) in a cell culture system. However, the *in vivo* role of WWP2 in mouse teeth remains unknown. To explore this, here we generated Wwp2 knockout (Wwp2 KO) mice. We found that molars in Wwp2 KO mice exhibited thinner dentin, widened predentin, and reduced numbers of dentinal tubules. In addition, expression of the odontoblast differentiation markers Dspp and Dmp1 was decreased in the odontoblast layers of Wwp2 KO mice. These findings demonstrate that WWP2 may facilitate odontoblast differentiation and dentinogenesis. Furthermore, we show for the first time that phosphatase and tensin homolog (PTEN), a tumor suppressor, is expressed in dental papilla cells and odontoblasts of mouse molars and acts as a negative regulator of odontoblastic differentiation. Further investigation indicated that PTEN is targeted by WWP2 for degradation during odontoblastic differentiation. We demonstrate PTEN physically interacts with and inhibits the transcriptional activity of KLF5 on Dspp and Dmp1. Finally, we found WWP2 was able to suppress the interaction between PTEN and KLF5, which diminished the inhibition effect of PTEN on KLF5. Taken together, this study confirms the essential role of WWP2 and the WWP2–PTEN–KLF5 signaling axis in odontoblast differentiation and dentinogenesis *in vivo*.

Odontoblasts are tooth-specific, terminally differentiated mesenchymal cells responsible for synthesis and secretion of collagens and noncollagenous proteins (1, 2). DSPP and DMP1 are two noncollagenous proteins and well recognized as important markers of odontoblast differentiation. They both contribute to the mineralization of predentin into dentin (3). And mineralized dentin plays essential roles in providing support for enamel and cementum and protecting dental pulp from outside stimuli (4). Therefore, unveiling the mechanisms of odontoblast differentiation and dentinogenesis is a hotspot in dental research field.

As a crucial form of posttranslational modifications, ubiquitination has been found to mediate various cellular activities such as cell differentiation and cell fate determination (5, 6). When being ubiquitinated, the lysines of the substrate are modified with one or more ubiquitin(s). Then the target protein can be sent for proteolysis, or its function or cellular localization will be altered. Ubiquitin ligases (also called E3 ligases; E3s) play a significant role in the ubiquitination process (7). WW domain–containing E3 Ubiquitin-protein ligase 2 (WWP2) is a multifunctional E3 ubiquitin ligase and participates in various developmental processes including osteogenesis and palatogenesis (8, 9, 10, 11, 12). Kruppel like factor 5 (KLF5) is an important odontoblast transcription factor. It is expressed in the odontoblast layer of mouse molars and incisors and can directly upregulate the expression of odontoblast marker genes, Dspp and Dmp1 (13, 14, 15). WWP2 has been reported to facilitate odontoblastic differentiation of mouse dental papilla cells (mDPCs) through augmenting KLF5 transcriptional activity on Dspp and Dmp1 in a nonproteolytic monoubiquitination manner (16). However, the *in vivo* role of WWP2 during odontoblast differentiation and dentinogenesis is still unknown.

Phosphatase and tensin homolog (PTEN) is a protein and lipid phosphatase that functions as a tumor suppressor. On the one hand, PTEN suppresses phosphatidylinositol 3 kinase/AKT signaling by hydrolyzing phosphatidylinositol (3,4,5)-trisphosphate to phosphatidylinositol (4,5)-bisphosphate, thus regulating various cellular behaviors including cell proliferation, differentiation, and migration. On the other hand, PTEN also mediates cell cycle, genomic stability, and gene expression in a phosphatidylinositol (3,4,5)-trisphosphate–independent manner (17, 18). PTEN has been found to participate in multiple developmental and regeneration processes, like prostate development, craniofacial development, T-cell lymphopoiesis, neurogenesis, and neural regeneration (19, 20, 21, 22, 23). Previously, PTEN was discovered to be present in the prospective preodontoblasts of dental papilla in human embryonic incisor tooth germs (24). However, both the expression pattern and function of PTEN during odontoblast differentiation remains unclear. PTEN has been recognized as a substrate of WWP2 for ubiquitination-dependent degradation (25, 26, 27). And a previous study has reported that Wwp2 knockout (Wwp2 KO) mice exhibit a phenotype similar to that of Pten transgenic mice with reduced sizes of the whole body and main organs (28). Nevertheless, the role of PTEN and whether it acts as a substrate of WWP2 during odontoblast differentiation needs further investigation.

In this study, Wwp2 KO mice were established. On account of the continuous growth of mouse incisors, molars were chosen for further investigation regarding the role of Wwp2 deletion in tooth development because their developmental processes are more similar to human teeth. Besides, the function of PTEN in odontoblastic differentiation of mDPCs and its potential mechanism was investigated. Our study confirmed that WWP2 mediates odontoblast differentiation and dentinogenesis by promoting Dspp and Dmp1 expression through the WWP2–PTEN–KLF5 axis.

## Results

### WWP2 promotes dentin formation *in vivo*

**Figure 1:**
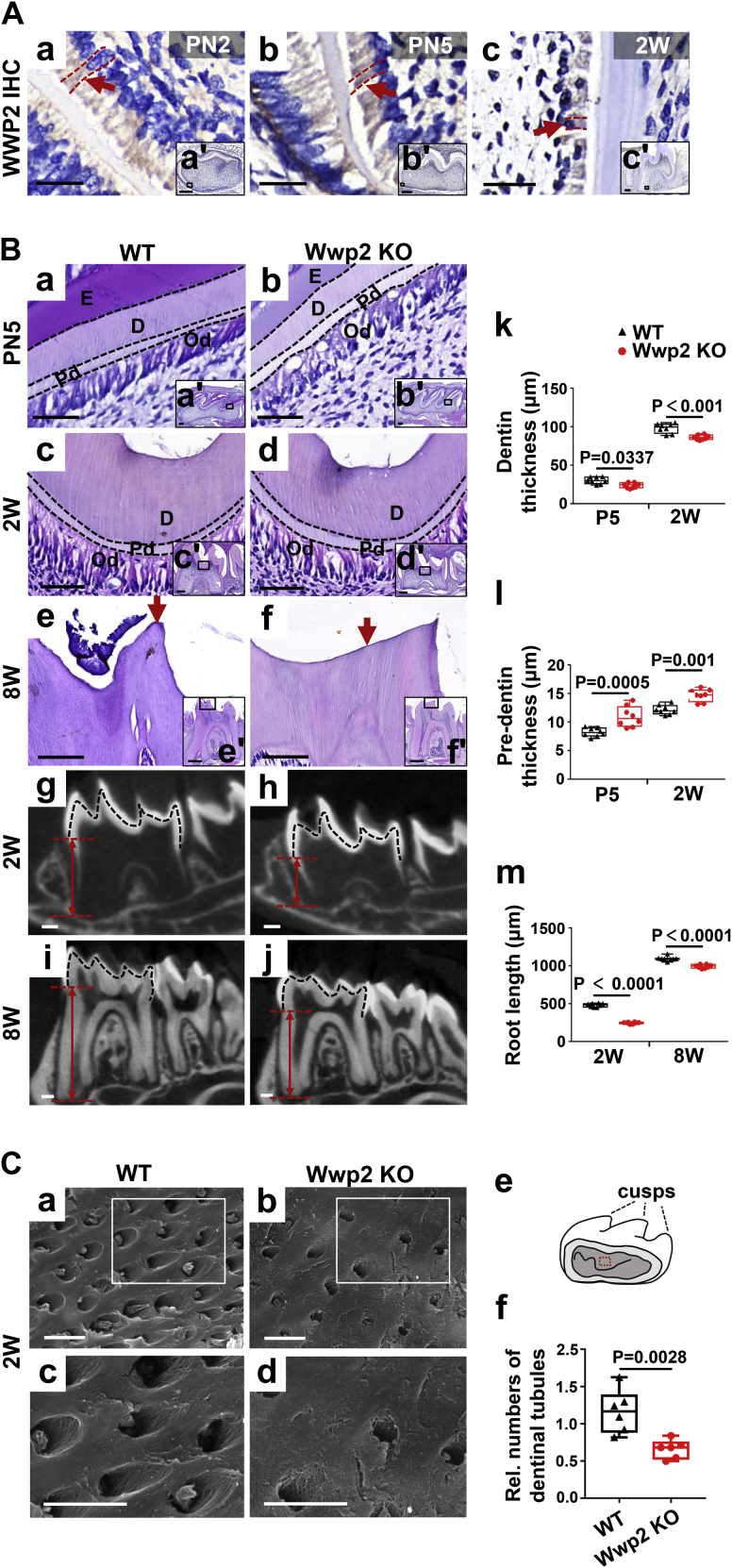
**WWP2 is essential for dentin formation.***A*, the expression of WWP2 in murine molars at PN2 (a and a’), PN5 (b and b’), and 2W (c and c’) shown by IHC. *Red arrows* show the positive WWP2 staining in odontoblasts. *Red dotted lines* manifest the boundaries of odontoblasts. (a–c) are higher magnifications of the rectangles in (a’–c’). *B*, H&E staining (a–f and a’–f’) and micro-CT analysis (g–j) of molars in WT and Wwp2 KO mice. In (k–m), quantitative analysis shows significant reduction of predentin and dentin thickness as well as root length in molars of Wwp2 KO mice. n = 8; Student’s *t* test; Error bars represent SD. To precisely calculate the dentin formation changes between WT and Wwp2 KO mice, the widths of predentin and dentin were measured in one H&E-stained section every five consecutive slices. *Black dotted lines* represent the boundaries of dentin and predentin in (a–d), and the boundaries between enamel and dentin (g–j). (a–f) are higher magnifications of the rectangles in (a’–f’). *Red arrows* show more severe cusp abrasion in molars of Wwp2 KO mice. *Red double arrows* mark the roots. *C*, scanning electron microscopy images show the dentinal tubules of molars at 2W in Wwp2 KO mice are less formed than in WT. (c, d) are higher magnifications of the rectangles in (a, b). The roof of pulp chamber marked by the red rectangle represents the site for scanning electron microscopy (e). The dentinal tubules in WT and Wwp2 KO mice at 2W were quantified (f). In (f), n = 8; Student’s *t* test; Error bars represent SD. The scale bars represent 5 μm for (*C*), 20 μm for (*A*) a, b, (*B*) a, a’, b, b’; 50 μm for (*A*) c, (*B*) c, d; 100 μm for (*B*) e, f; 200 μm for (*A*) a’–c’ and (*B*) c’, d’, g–j, 500 μm for e’, f’. D, dentin; E, enamel; Od, odontoblast; Pd, predentin; Rel., relative.

The expression pattern of WWP2 during early molar development has been reported in our previous study (16). Here, murine molars at later stages from postnatal 2 days (PN2) to 2 weeks (2W) were subjected to immunohistochemistry (IHC). The results showed that WWP2 was distinctly expressed in the odontoblast layer (Fig. 1*A*). The negative control of IHC was shown in Fig. S2.

The *in vivo* role of WWP2 in regulating odontoblast differentiation and dentinogenesis was further investigated by Wwp2 KO mice. Knockout of Wwp2 was verified by genotyping, Western blot, and IHC (Fig. S1, *B*–*D*). Interestingly, hematoxylin and eosin (H&E) staining revealed reduced widths of the mineralized dentin but increased widths of the nonmineralized predentin in Wwp2 KO mice at PN5 and 2W (Fig. 1*B* a–d, k and l). Meanwhile, ablation of Wwp2 led to more abrasion at the molar cusps (Fig. 1*B* e and f). Micro–computed tomography verified shorter roots in the molars of Wwp2 KO mice (Fig. 1*B* g–j and m). Besides, scanning electron microscopy of molars at 2W displayed less numbers of dentinal tubules in Wwp2 KO mice compared with the wildtype (WT) group (Fig. 1*C* a–f). Heterozygous mice did not show these defects (data not shown).

### WWP2 is essential for the differentiation and mineralization ability of odontoblasts

**Figure 2:**
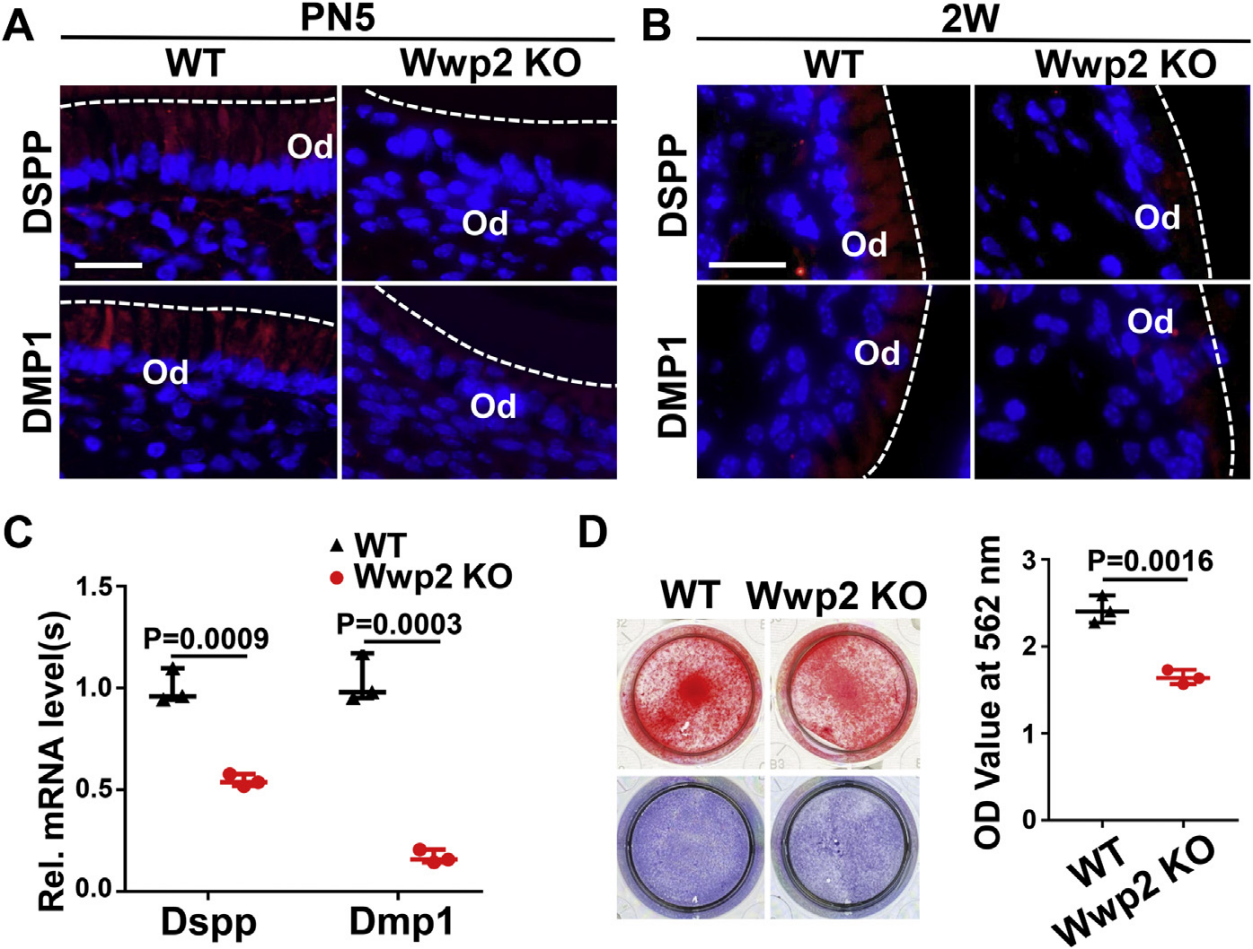
**WWP2 promotes the differentiation of odontoblasts.***A* and *B*, immunofluorescence staining of DSPP and DMP1 in molars of WT and Wwp2 KO mice at PN5 (*A*) and 2W (*B*). *Red fluorescence* shows the positive expression of DSPP or DMP1, while *blue fluorescence* shows the nucleus. *C*, the mRNA levels of Dspp and Dmp1 were significantly reduced in differentiation-induced Wwp2 KO mDPCs. n = 3; Student’s *t* test; Error bars represent SD. *D*, alizarin red S and alkaline phosphatase staining of WT and Wwp2 KO differentiation-induced mDPCs. For alizarin red S, the mDPCs were cultured in differentiation medium (DM) for 7 days. Ten percent cetylpyridinium chloride was used for harvesting the stained cells, and the absorbance values were measured at 562 nm. For alkaline phosphatase staining, the mDPCs were cultured in DM for 5 days. n = 3; Student’s *t* test; Error bars represent SD. The scale bars represent 20 μm in (*A*) and (*B*). Rel., relative.

To verify the *in vivo* role of WWP2 in odontoblast differentiation, the expression changes of odontoblast markers, DSPP and DMP1, were investigated by immunofluorescence (IF). The results showed that the expression levels of both markers were decreased in Wwp2 KO odontoblast layers compared with the WT group at PN5 and 2W (Fig. 2, *A* and *B*). The negative control of IF was shown in Fig. S3.

mDPCs from molars in both WT and Wwp2 KO mice were isolated and cultured in differentiation medium. Quantitative real-time PCR experiments showed that the mRNA levels of Dspp and Dmp1 were declined in Wwp2 KO cells (Fig. 2*C*). The mineralization ability of odontoblastic cells was assessed by alizarin red S (ARS) staining and alkaline phosphatase (ALP) assays (Fig. 2*D*). As expected, knockout of Wwp2 led to a decrease of mineralized nodule formation. ALP activity was also hampered by Wwp2 deficiency (Fig. 2*D*).

### PTEN acts as an inhibitor for odontoblastic differentiation

**Figure 3:**
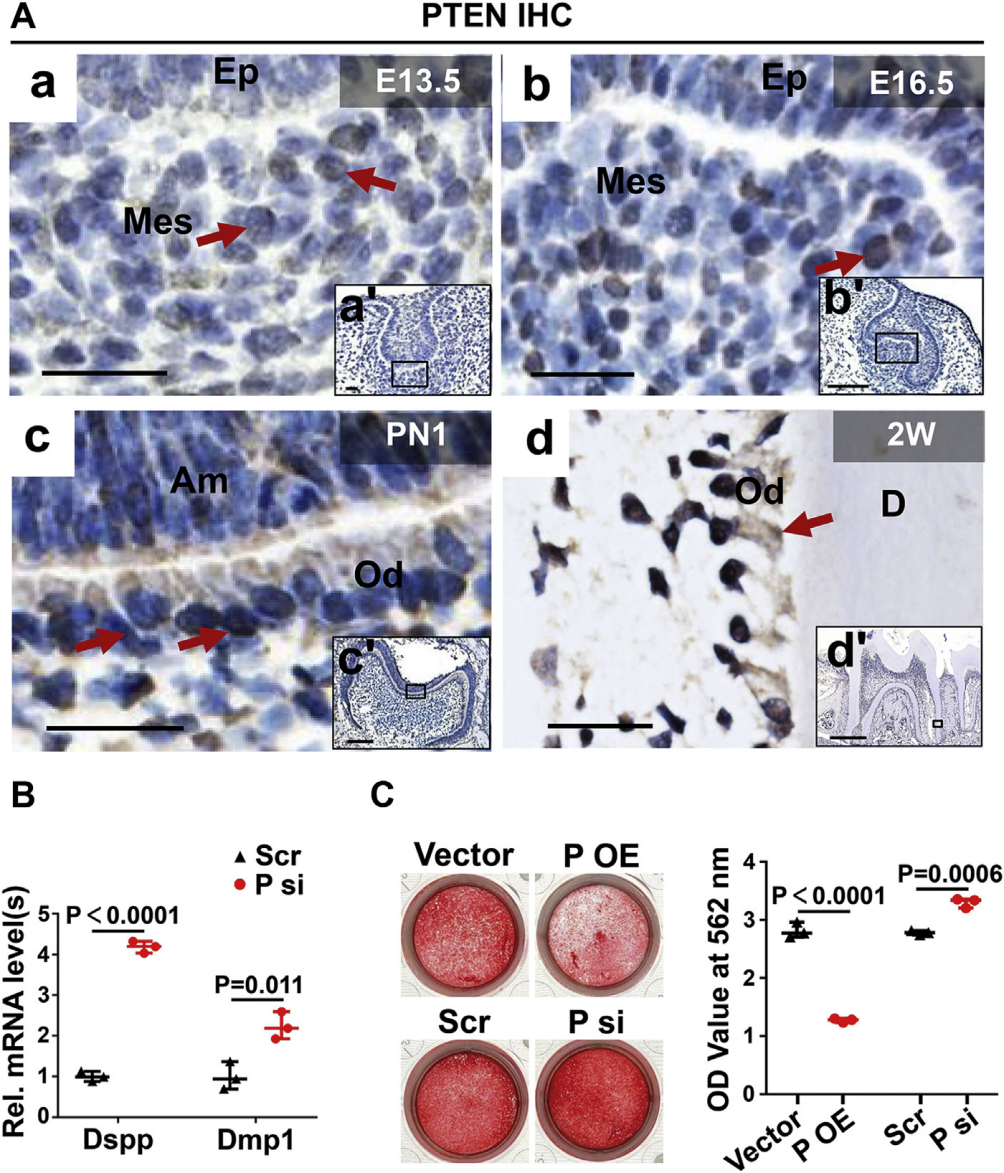
**PTEN is expressed during tooth development and suppresses odontoblastic differentiation.***A*, the expression pattern of PTEN during tooth development detected by immunohistochemistry. (a–d) are higher magnifications of the rectangles in (a’–d’). *Red arrows* point to the positive signals of PTEN. *B*, the effects of Pten knockdown on the mRNA levels of Dspp and Dmp1. The mDPCs were cultured in DM for 7 days. n = 3; Student’s *t* test; Error bars represent SD. *C*, the effects of Pten knockdown or overexpression on the formation of mineralized nodules. The mDPCs were cultured in DM for 7 days and then were stained by alizarin red S. Ten percent cetylpyridinium chloride was used for harvesting the stained cells, and the absorbance values were measured at 562 nm. n = 3; Student’s *t* test; Error bars represent SD. The scale bars represent 20 μm for (*A*) a–d and a’, 100 μm for (*A*) b’–c’, and 500 μm for (*A*) d’. Am, ameloblast; D, dentin; Ep, epithelium; Mes, mesenchyme; Od, odontoblast; OE, overexpression; P, Pten; Scr, scramble; si, knockdown; Rel., relative.

To observe the expression pattern of PTEN in dental mesenchyme, IHC was performed in murine molars. The results showed that PTEN was positively stained in the dental papilla cells at E13.5 and E16.5 (Fig. 3*A* a and b). At PN1 and 2W, PTEN was expressed in the odontoblast layer (Fig. 3*A* c and d). Real-time PCR and ARS experiments showed that knockdown of Pten augmented the mRNA levels of Dspp and Dmp1 (Fig. 3*B*) as well as the formation of mineralized nodules (Fig. 3*C*). On the contrary, overexpression of Pten inhibited the formation of mineralized nodules (Fig. 3*C*). These results demonstrated that PTEN plays a negative role in odontoblastic differentiation.

### WWP2 physically interacts with PTEN in mouse molars

**Figure 4:**
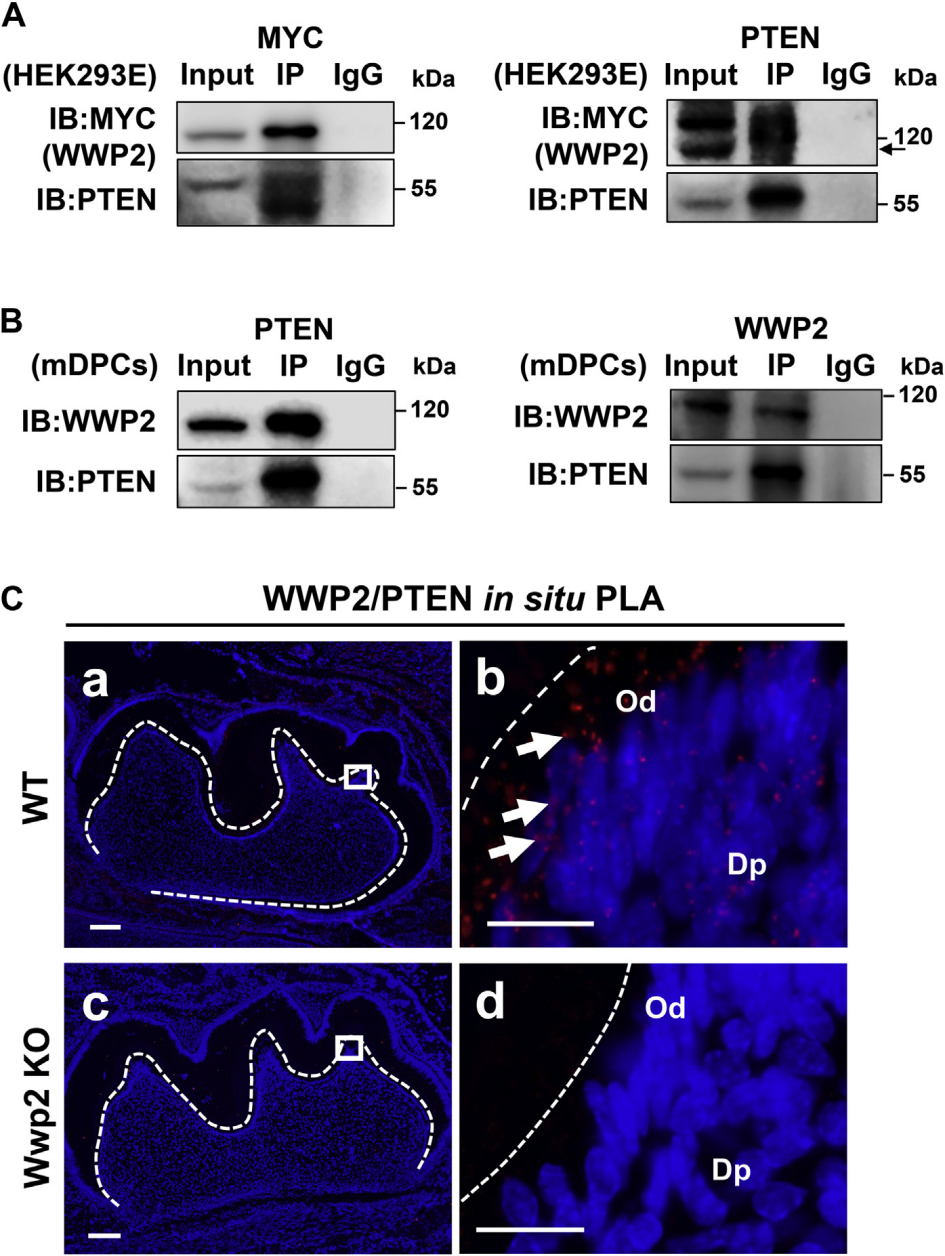
**WWP2 physically interacts with PTEN in mouse molars.***A*, coimmunoprecipitation assays show the interaction between WWP2 and PTEN in HEK293E cells (overexpressed proteins). The *arrow* shows the indicated bands. *B*, coimmunoprecipitation assays show the interaction between WWP2 and PTEN in mDPCs (endogenous proteins). *C*, *in vivo* interaction between PTEN and WWP2 in PN5 molars of WT and Wwp2 KO mice revealed by *in situ* proximity ligation assay. b, d are higher magnifications of the rectangles in a, c. The scale bars represent 100 μm for (*C*) a and 20 μm for (*C*) b. Dp, dental papilla; Od, odontoblast.

WWP2 is expressed in the odontoblast layer and the underlying dental papilla cells (16). The identical expression pattern between PTEN and WWP2 suggests that they may have physical interaction. First, coimmunoprecipitation (co-IP) assays were performed. Overexpressed WWP2 and PTEN were shown to interact with each other in Human embryonic kidney 293E cell lines (HEK293E) cells (Fig. 4*A*). Besides, the interaction between endogenous WWP2 and PTEN in mDPCs was also observed (Fig. 4*B*). As expected, *in situ* proximity ligation assays (PLAs) further confirmed their interaction in odontoblasts and dental papilla cells in murine molars (Fig. 4*C* a and b). Deletion of Wwp2 abolished this interaction effect *in vivo* (Fig. 4*C* c and d).

### WWP2 regulates odontoblastic differentiation by promoting PTEN proteolysis

**Figure 5:**
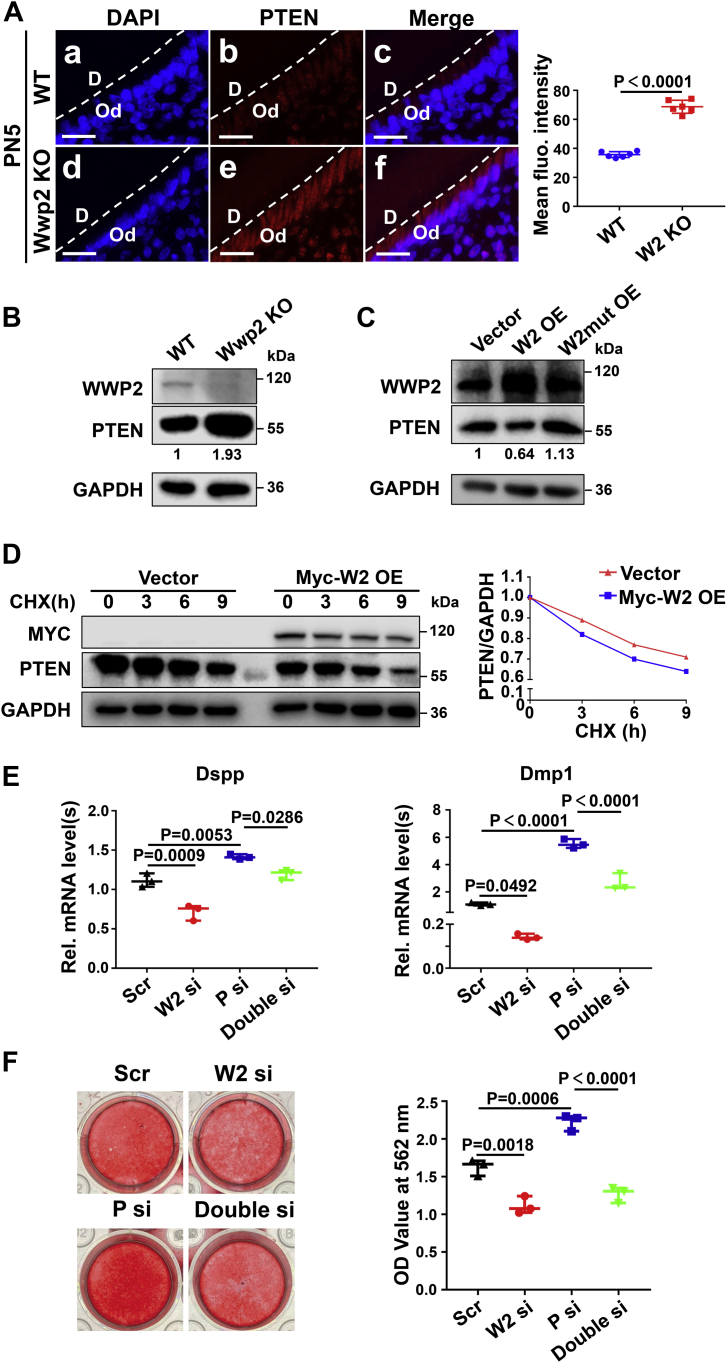
**WWP2 regulates odontoblastic differentiation by promoting PTEN proteolysis.***A*, increased PTEN expression in the odontoblast layer of Wwp2 KO mice revealed by immunofluorescence. The mean fluorescence intensity of PTEN was quantified by Image J software. n = 6; Student’s *t* test; Error bars represent SD. *B*, elevated PTEN level in differentiation-induced Wwp2 KO mDPCs shown by Western blot. *C*, changes of PTEN expression levels when Wwp2 or Wwp2 ligase activity–dead mutant was overexpressed. *D*, cycloheximide (CHX) assay showed that overexpression of Wwp2 accelerated the degradation rate of PTEN. CHX was applied to the transfected mDPCs or the untreated cells for 0, 3, 6, 9 h. The cell lysates were harvested for Western blot analysis. *E*, Dspp and Dmp1 mRNA levels when Wwp2 and Pten were knocked down separately or simultaneously. The transfected mDPCs were cultured in DM for 7 days, and real-time PCR assays were performed. n = 3; Student’s *t* test; Error bars represent SD. *F*, the effect of WWP2 and PTEN on the mineralized nodule formation. The transfected mDPCs were cultured in DM for 7 days. Alizarin red S was applied and the stained cells were harvested using 10% cetylpyridinium chloride. The absorbance values were measured at 562 nm. n = 3; Student’s *t* test; Error bars represent SD. The scale bars represent 20 μm for (*A*). D, dentin; fluo., fluorescence; Od, odontoblast; OE, overexpression; P, Pten; Rel., relative; Scr, scramble; si, knockdown; W2, Wwp2; W2mut, Wwp2 ligase activity–dead mutant.

Next, the proteolysis effect of WWP2 on PTEN was investigated *in vivo* and *in vitro*. IF revealed that ablation of WWP2 decreased degradation of PTEN and stabilized PTEN protein level at the odontoblast layer of mouse molars *in vivo* (Figs. 5*A* and S4, *A* and *B*). Western blot assay also showed that knockout of Wwp2 upregulated the protein level of PTEN (Fig. 5*B*). Overexpression of WWP2 led to decreased PTEN level, while overexpression of ligase-dead mutant WWP2 (WWP2C838A, in which cysteine 838 of WWP2 was mutated to alanine) did not show this effect (Figs. 5*C* and S5, *A* and *A*’). The regulation of WWP2 on the transcription level of Pten was excluded (Fig. S6*A*). Furthermore, it was demonstrated that overexpressed WWP2 accelerated the degradation rate of PTEN by using cycloheximide (a protein synthesis inhibitor) (Figs. 5*D* and S5, *B* and *B*’). The decreased differentiation and mineralization ability of mDPCs due to Wwp2 knockdown can be rescued by Pten knockdown (Fig. 5, *E* and *F*). Therefore, WWP2 promotes odontoblastic differentiation through targeting PTEN for degradation.

### WWP2 inhibits the suppression effect of PTEN on KLF5 transcriptional activity

**Figure 6:**
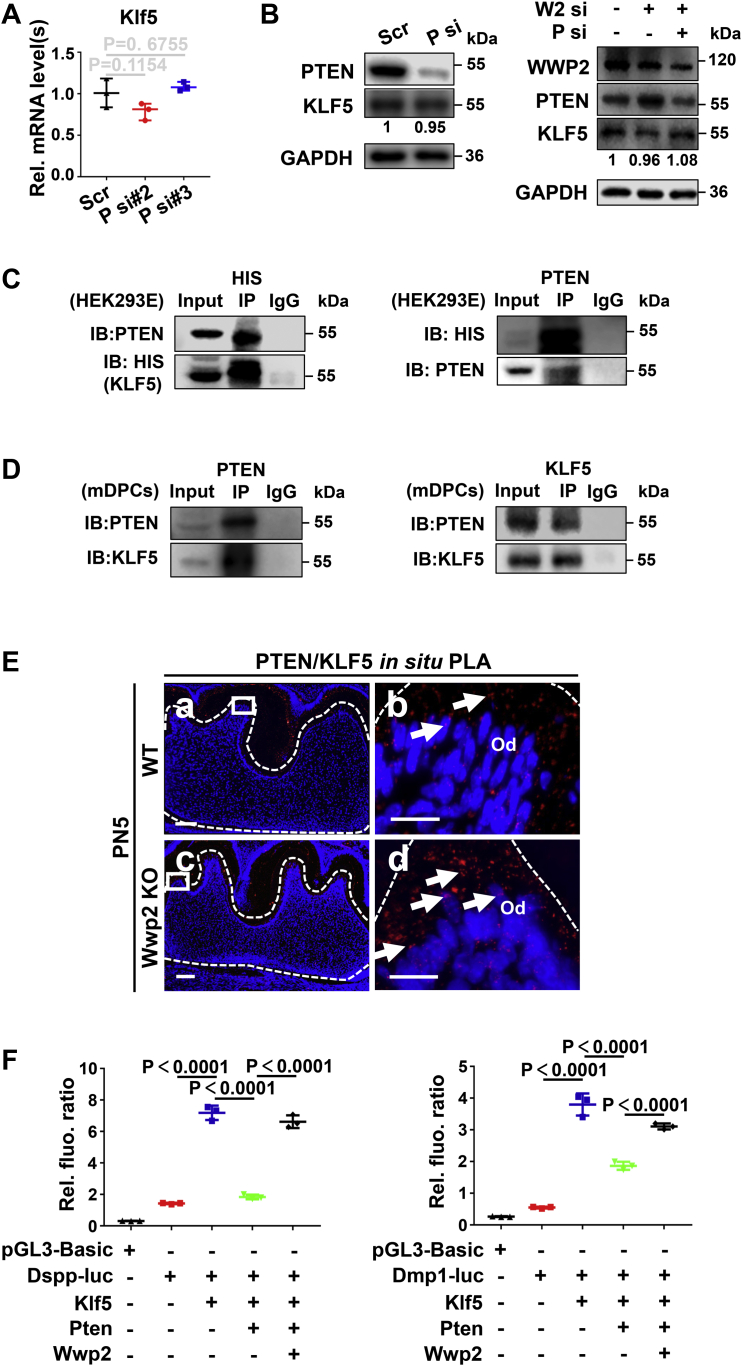
**WWP2 inhibits the suppression effect of PTEN on KLF5 transcriptional activity.***A*, the effect of PTEN on Klf5 mRNA levels investigated by real-time PCR. n = 3; Student’s *t* test; Error bars represent SD. *B*, the effect of PTEN and WWP2 on KLF5 protein levels revealed by Western blot. *C*, coimmunoprecipitation assays show the interaction between PTEN and KLF5 in HEK293E cells (overexpressed proteins). *D*, coimmunoprecipitation assays show the interaction between PTEN and KLF5 in mDPCs (endogenous proteins). *E*, the interaction between KLF5 and PTEN *in vivo* shown by *in situ* proximity ligation assay. b and d are higher magnifications of the rectangles in a and c. The *arrows* show the positive signals for KLF5-PTEN interaction. *F*, the function of PTEN and WWP2 on KLF5-mediated Dspp- and Dmp1-luciferase reporter activities by dual luciferase assays. The mDPCs were harvested after 48-h transfection. n = 3; ANOVA; error bars represent SD. The scale bars represent 100 μm for (*C*) a, c; 20 μm for (*C*) b, d. fluo., fluorescence; luc, luciferase plasmid; Od, odontoblast; P, Pten; Rel., relative; Scr, scramble; si, knockdown; Ub, ubiquitin; W2, Wwp2.

In addition to PTEN, KLF5 as an important transcription factor for odontoblast differentiation is another substrate of WWP2 during odontoblastic differentiation (16), but whether there is a cross talk between PTEN and KLF5 is not clear. Knockdown and overexpression experiments demonstrated that Pten could not influence the mRNA and protein levels of Klf5 (Fig. 6, *A* and *B*). However, co-IP assays illustrated that PTEN bound to KLF5 in HEK293E cells (Fig. 6*C*) and mDPCs (Fig. 6*D*). *In situ* PLA further revealed the *in vivo* interaction between PTEN and KLF5 in the odontoblast layer of PN5 molars (Fig. 6*E*). Dual luciferase reporter assay was applied to detect whether PTEN influenced the transcriptional activity of KLF5. The results showed that concomitant overexpression of Pten inhibited the upregulated luciferase activity of both Dspp and Dmp1 promoters by overexpression of Klf5 alone (Fig. 6*F*). Notably, although WWP2 had no effect on the mRNA or protein level of KLF5 (Figs. 6*B* and S7, *A* and *B*), overexpression of WWP2 was able to rescue the impediment of PTEN on KLF5 transcriptional activity (Fig. 6*F*).

## Discussion

In the cell culture system we have previously found WWP2 can monoubiquitinate KLF5 and promote the transcriptional activity of KLF5 on Dspp and Dmp1. In this study, it was found that depletion of WWP2 impacted odontoblast differentiation and dentin formation. PTEN is a well-known tumor suppressor. It regulates various cellular activities of diverse cell types (17, 18). In this study, PTEN was found to negatively regulate odontoblastic differentiation. Some studies have shown that the E3 ligase WWP2 is able to target PTEN for ubiquitination-dependent proteolysis (26, 27, 28, 29). This also applies to the odontoblastic differentiation process. Further investigation revealed that WWP2 bound to and degraded PTEN in odontoblasts, thus relieving the suppression effect of PTEN on the transcriptional activity of KLF5.

WWP2 is an active player in some developmental processes. A previous study uncovered that WWP2 was responsible for the proper development of cortical neurons, in coordination with its family member WWP1 (11). WWP2 was also revealed to monoubiquitinate RUNX2 and promote osteogenic differentiation (9). Similarly, in a cell culture system, WWP2 was illustrated to promote odontoblastic differentiation by monoubiquitinating KLF5 and elevating the transcription levels of its downstream genes, Dspp and Dmp1 (16). In the present study, Wwp2 KO mice were generated by the CRISPR-Cas9 technique for further investigation of the *in vivo* function of WWP2 in odontoblast differentiation and dentinogenesis. Notably, Wwp2 KO mice exhibited the phenotype with thinner dentin layer, widened predentin layer, as well as shorter roots. Meanwhile, the dentinal tubules of molars in Wwp2 KO mice were less formed shown by scanning electron microscopy. Therefore, WWP2 plays a positive role for odontoblast differentiation and dentin formation in mice. Early cusp abrasion in the molars of Wwp2 KO mice suggests that ablation of Wwp2 led to declined mechanical property of teeth. Intriguingly, previous studies have shown that depletion of either Dspp or Dmp1 led to widened predentin and thinner dentin layer (30, 31, 32), which resembled the phenotypes of Wwp2 KO mice in our study. Besides, Dmp1 KO mice showed a decrease in the amount of dentinal tubules (31), which was also similar to Wwp2 KO mice in our study.

Then the expression levels of DSPP and DMP1 were detected. It was demonstrated that the levels of DSPP and DMP1 proteins were decreased in the molars of Wwp2 KO mice by IF. In cultured mDPCs, Wwp2 deletion led to a reduction of Dspp and Dmp1 mRNA levels, a decrease of mineralized nodule formation and ALP activity. These results were consistent with our previous *in vitro* study (16), further demonstrating that WWP2 is indispensable for proper differentiation and mineralization ability of odontoblasts.

PTEN has been found to mediate multiple cellular processes (18), but its expression pattern and function in tooth development remained unclear. In this study, PTEN was found positively stained in murine molars at different developmental stages (E13.5, E16.5, PN1 and 2W), indicating that PTEN may participate in tooth development. The function of PTEN in odontoblastic differentiation was further explored by a series of experiments, which verified that PTEN negatively regulates odontoblastic differentiation. A lot of evidences have revealed that PTEN is able to mediate the differentiation process of many cell types in positive or negative ways. For example, knockout of Pten in neural crest cells stimulated their osteoblastic differentiation (21). Knockdown of Pten markedly promoted neuronal cell differentiation (33). However, there are also some researches showing positive roles of PTEN on cell differentiation. Under BMP2 stimulation, PTEN was upregulated and then facilitated the differentiation of hair follicle stem cells by inducing autophagy (34). And in terms of immune cells, PTEN promoted T helper 17 cell differentiation through inhibiting IL-2 expression (35). Therefore, the function of PTEN in controlling cell differentiation possibly depends on cell types and circumstances.

Regulation of PTEN, including posttranslational modification by E3 ligases, is significant for the protein level and phosphatase activity of PTEN (36). WWP2 has been identified to ubiquitinate and regulate PTEN in prostate cancer cells (26). Therefore, it was wondered whether WWP2 also targeted PTEN for ubiquitination-dependent degradation in odontoblasts. The interaction between WWP2 and PTEN is a prerequisite for degradation of PTEN by WWP2, and their interaction *in vitro* and *in vivo* was verified by co-IP and *in situ* PLA, respectively. Wwp2 deletion did lead to an increase of PTEN protein level *in vitro* and *in vivo*. Overexpression of WWP2 prompted the degradation of PTEN protein, while overexpression of the ligase activity–dead WWP2 did not have this effect, suggesting the degradation of PTEN is dependent on the ligase activity of WWP2. Cycloheximide assays further confirmed that WWP2 promoted PTEN proteolysis. Concomitant knockdown of Pten rescued the decreased differentiation of mDPCs caused by Wwp2 knockdown, suggesting that upregulated PTEN accounts for the inhibition effects of Wwp2 knockdown. All these results demonstrated that WWP2 promotes odontoblast differentiation and dentinogenesis through targeting PTEN for degradation.

Since our previous study found that WWP2 was able to monoubiquitinate KLF5 and activate its transcriptional activity (16), we wondered whether a cross talk between PTEN and KLF5 existed during odontoblastic differentiation. The regulation of the transcriptional and translational levels of KLF5 by PTEN was first excluded. Interestingly, PTEN was found to bind with KLF5 in both isolated cells and molars. And their interaction increased in the molars of Wwp2 KO mice, which may be attributed to the elevated PTEN protein level by Wwp2 deletion. PTEN was able to inhibit the transcriptional activity of KLF5 on Dspp and Dmp1. As expected, Wwp2 overexpression suppressed the inhibition effects of PTEN on KLF5 transcriptional activity, which can be partially explained by the degradation of PTEN by WWP2. Similarly, PTEN has been found to participate in regulating transcriptional activity of other transcription factors, like Forkhead transcription factors (FOXO) (37). PTEN has been reported to exert its function through phosphatidylinositol 3 kinase/ATK-dependent or -independent pathways (38, 39). Whether PTEN also influences the transcriptional activity of KLF5 through these pathways needs further investigation.

**Figure 7:**
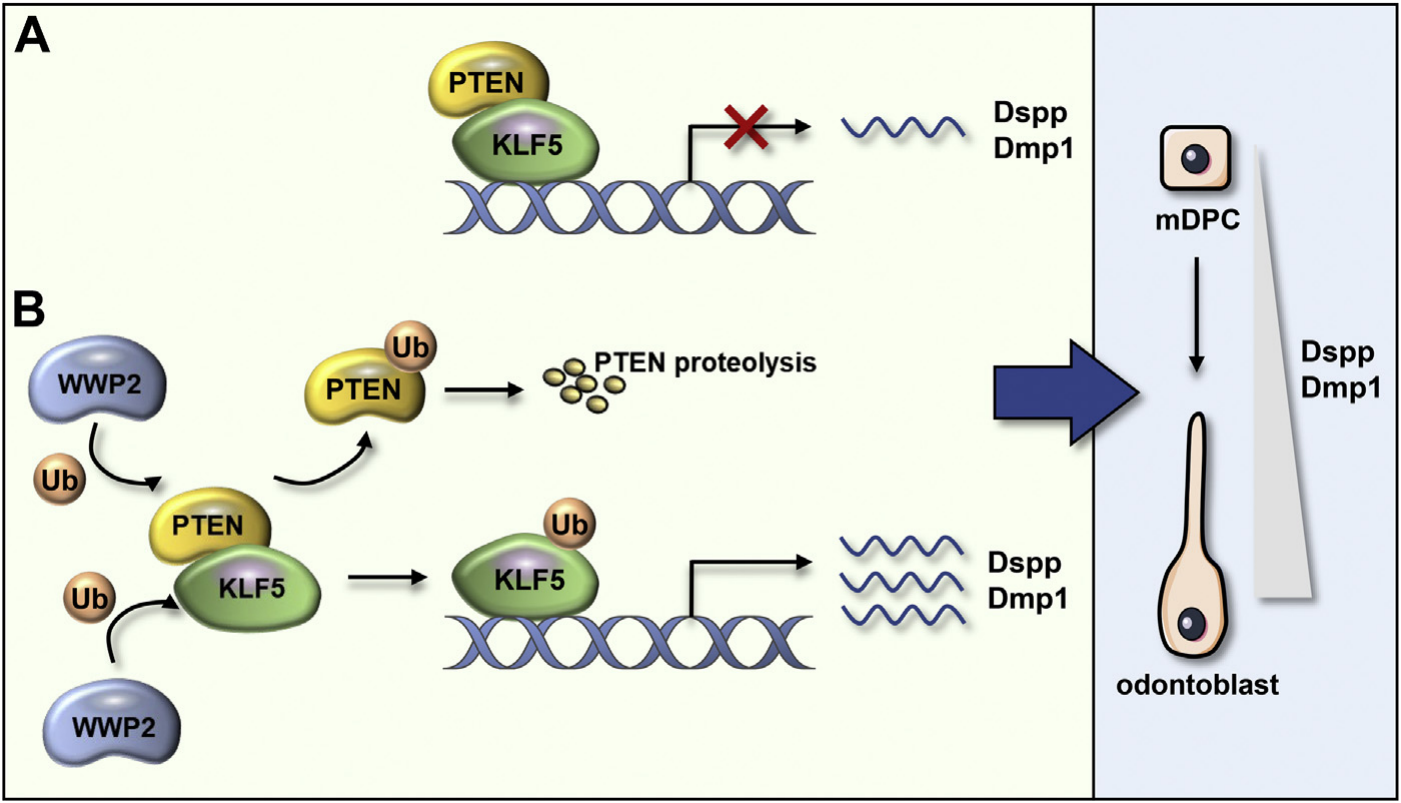
**The schema of WWP2–PTEN–KLF5 interaction, which regulates odontoblast differentiation and dentinogenesis by mediating Dspp and Dmp1 transcription levels.***A*, PTEN interacts with KLF5 and suppresses the transcriptional activity of KLF5 on Dspp and Dmp1. *B*, WWP2 promotes the degradation of PTEN and antagonizes the suppression on KLF5 transcriptional activity by PTEN. Meanwhile, WWP2 monoubiquitinates KLF5 and enhances the transcriptional activity of KLF5 on Dspp and Dmp1.

In summary, molars in Wwp2 KO mice show the phenotype with thinner dentin, widened predentin, and shorter roots. The dentinal tubules of molars in Wwp2 KO mice are less formed and disordered compared with the WT group. PTEN is expressed in dental mesenchymal cells and odontoblasts during tooth development. PTEN can interact with KLF5 and suppress the transcriptional activity of KLF5 on Dspp and Dmp1, thus playing a negative role for odontoblastic differentiation of mDPCs (Fig. 7*A*). WWP2 can target PTEN for degradation and consequently antagonize the suppression on KLF5 transcriptional activity by PTEN. Besides, WWP2 interacts with and monoubiquitinates KLF5, thus enhancing the transcriptional activity of KLF5 on Dspp and Dmp1. WWP2 mediates odontoblast differentiation and dentinogenesis by promoting the expression levels of Dspp and Dmp1 through the WWP2–PTEN–KLF5 axis (Fig. 7*B*) (16). The above mechanism can well explain the defects in the differentiation and mineralization ability of Wwp2 KO odontoblasts. Previous studies have reported that mesenchymal cell proliferation is essential for root development size (40, 41); whether cell proliferation alteration exists and contributes to shortened roots in Wwp2 KO mice needs further investigation. Although mice are ideal genetic animal models, they are different from humans for having only one dentition; more investigation on two-dentition animal models is needed.

## Experimental procedures

### Mice and tissue collection

All mice were raised in the specific pathogen-free facility with a 12-h light/dark cycle in ventilated cages in groups not surpassing four animals. Mice were fed by standard rodent chow from Huafukang (Cat No: 1032). All animal experiments were approved by the Animal Welfare and Ethics Committee of the School and Hospital of Stomatology at Wuhan University (S07919010A).

Wwp2 KO mice of C57BL/6J background were established at Gempharmatech (Project NO: GPS00001611). The knockout strategy using the CRISPR-Cas9 technology was described in Fig. S1*A*. The primers for genotyping of WT and Wwp2 KO mice are listed in Table S1 and were synthesized at Sangon. Other information, such as ages of samples and replicates, is shown in the figure legends and Table S2. The KO mice were viable and fertile. The birth of the KO mice was conformed to Mendel's law of inheritance. In all the animal experiments, both male and female mice were used.

All mice were sacrificed using carbon dioxide inhalation. The mandibles were isolated. After being fixed with 4% paraformaldehyde at 4 °C overnight, the mandibles were observed by micro–computed tomography. For paraffin slicing, the tissues were decalcified with 10% ethylenediaminetetraacetic acid, then dehydrated and embedded with paraffin.

### Histological staining

For histological staining, the 5-μm slices were deparaffinized and rehydrated. H&E staining was performed with hematoxylin and eosin dyes. IHC was performed using the HRP-Polymer anti-Rabbit IHC Kit (MaxVision) according to the manufacturer’s instructions. The primary antibodies of WWP2 (Proteintech) and PTEN (Abclonal) were each applied at a 1:200 concentration. After being stained, the slices were mounted by neutral balsam.

For IF, the concentrations of antibodies applied were as follows: DSPP (1:200; Novus), DMP1 (1:200; Abclonal), PTEN (1:200; Abclonal), and Alexa Fluor Red 594 Donkey anti Rabbit IgG (1:400; Antgene). After being stained, the slices were mounted by DAPI.

### Cell culture and cytologic staining

The primary mDPCs were isolated from the first mandibular molars of neonatal mice and seeded into cell plates or dishes after 0.25% trypsin digestion for 10 min as described (16). HEK293E and mDPCs were cultured in Dulbecco’s modified Eagle’s medium (HyClone) supplemented with 10% fetal bovine serum (Gibco). mDPCs were induced for odontoblastic cells by differentiation medium supplemented with 10% fetal bovine serum, 10 mM β-glycerophosphate, 10 nM dexamethasone, and 50 μg/ml ascorbic acid (Sigma-Aldrich). For cytologic staining, cells were fixed with 4% polyoxymethylene at 4 °C for 15 min. Then ALP staining was performed using the BCIP/NBT Alkaline Phosphatase Kit (Beyotime) and ARS staining was applied with 0.5% ARS solution (pH = 4.5) (Aladdin). For ARS, 10% cetylpyridinium chloride was used for harvesting the stained cells, and absorbance values were measured at 562 nm by the spectrophotometer and normalized to the total protein concentration per well.

### *In situ* PLA

To histologically visualize the interaction between PTEN and KLF5 (or WWP2) in mouse molars, *in situ* PLA was performed using the Duolink kit (Sigma-Aldrich) as described in our previous study (42). In brief, the slices were deparaffinized, rehydrated, and antigen-retrieved, then blocked and incubated with anti-PTEN (mouse monoclonal; Proteintech) antibody together with either anti-KLF5 (rabbit polyclonal; Proteintech) or anti-WWP2 (rabbit polyclonal; Proteintech) antibody at 4 °C overnight. Afterward, the slices were washed and incubated with secondary antibodies conjugated with oligonucleotides. Slices subsequently went through ligation reaction as well as amplification reaction. Finally, slices were mounted with DAPI and observed under fluorescence microscope (Olympus Digital Camera).

### Coimmunoprecipitation

mDPCs or transfected HEK293E cells were rinsed with PBS and lysed. The cell lysates were collected into the microcentrifuge tubes and shaken gently at 4 °C for 30 min. The supernatant was removed to new microcentrifuge tubes after centrifugation. Co-IP assays were performed as described (16). The primary antibodies or the negative control IgG were as follows: WWP2 (Abcam), KLF5 (Proteintech), PTEN (CST), MYC (Proteintech), HIS (Abcam), IgG (Antgene). After being mixed with the primary antibodies or the negative control IgG, the samples were rotated gently at 4 °C overnight. Then the protein A/G magnetic beads (Bimake) were added to the samples and the mixtures were rotated gently for 1 h. After being washed, the supernatant was discarded and the magnetic beads were resuspended with SDS. The samples were denatured at 95 °C for 15 min and analyzed by Western blotting.

### Small interfering RNA and plasmid transfection

The siRNAs of Wwp2 and Pten were purchased from Genepharma. The information of overexpression plasmids is listed in Table S3. mDPCs or HEK293E cells were seeded into plates or dishes and were transfected with siRNAs or plasmids with Lipofectamine 3000 (Invitrogen) according to the manufacturer’s instructions. The negative control scrambled siRNAs and the vector plasmids were transfected as the control groups.

### Western blot and real-time PCR

The cells were lysed by NP-40 (Beyotime) containing 1/100 protease inhibitor Cocktail (MedChemExpress) for 15 min at 4 °C. After supersonic lysis and centrifugation, the supernatant was obtained and the protein concentration was measured using the Bicinchoninic Acid Protein Assay Kit (Thermo). Then the protein samples were mixed with SDS and denatured at 95 °C for 15 min. The samples were fractionated by 10% SDS-PAGE and transferred to Trans-blot membranes (Roche). The membranes were blocked by 5% skim milk for 1 h at room temperature and incubated with the primary antibodies as follows: WWP2 (1:1000; Abcam), KLF5 (1:1000; Proteintech), GAPDH (1:10,000; Proteintech), HIS (1:1000; Abcam), MYC (1:1000; Abcam), and PTEN (1:1000; Abclonal). After being incubated with secondary antibodies, the membranes were detected by ECL solution (Advansta).

Real-time PCR was carried out using fluorescent quantitation PCR mix from Vazyme in accordance with the manufacturer’s instructions. The primers were synthesized at Sangon. The primer sequences are listed in the Table S4.

### Scanning electron microscopy

Mandibular molars of Wwp2 KO and WT mice at 2W were fractured vertically from the central fossae and rapidly fixed in 2.5% glutaraldehyde at 4 °C overnight. After being washed, the samples were treated by acid etching by 30% phosphoric acid for 15 s and 5.25% sodium hypochlorite for 4 to 5 min. Then the tissues were washed and dehydrated in an ascending alcohol series. After that, the samples were dried by air and were sputter coated with gold for scanning electron microscopy (TESCAN VEGA3 LMU) (43).

### Dual luciferase reporter assay

The dual luciferase reporter assay was performed as described (16). Briefly, HEK293E cells seeded into 24-well plates were cotransfected with overexpression plasmids. The plasmids of each group are listed in the figures. pRL-TK was applied as internal reference. After being transfected for 48 h, the relative fluorescence ratio of the cells was detected using the kit of Dual-Luciferase Reporter Assay System (Promega) and the Glomax Luminometer (Promega).

### Statistical analysis

Experiments were independently repeated three times. Student’s *t* test (two tailed) was used to analyze the statistically significant differences of two groups, and one-way analysis of variance (ANOVA) followed by Tukey’s test was applied for three or more groups. All statistical analyses were performed by GraphPad Prism software (version 8.0). The results are represented as means ± standard deviation (means ± SD). *p* < 0.05 is defined as statistically significant.

## Data availability

All data in this study are contained within the article and the supplemental materials.

## Supporting information

This article contains supporting information (13, 16).

## Conflict of interest

The authors declare that they have no conflicts of interest with the contents of this article.
