# Supplementary material for: A WWP2–PTEN–KLF5 signaling axis regulates odontoblast differentiation and dentinogenesis in mice
Source: J Biol Chem. 2022 Jul 1;298(8):102220. doi: 10.1016/j.jbc.2022.102220 (PMC9358474; doi:10.1016/j.jbc.2022.102220)
Supplement: Supplemental Tables S1–S4 and Figures S1–S7 [file mmc1.docx]

**A WWP2-PTEN-KLF5 signaling axis regulates odontoblast differentiation and dentinogenesis in mice**

**Running Title: A WWP2-PTEN-KLF5 axis regulates dentinogenesis *in vivo***

Jing Fu^1,2^, Xiaobo Zhang^1,2^, Huiwen Zheng^1,2^, Guobin Yang^1^, Zhi Chen^1^, Guohua Yuan^1,2,*^

^1^The State Key Laboratory Breeding Base of Basic Science of Stomatology and Key Laboratory for Oral Biomedicine of Ministry of Education, School and Hospital of Stomatology, Wuhan University, Wuhan, China.

^2^Frontier Science Center for Immunology and Metabolism, Wuhan University, Wuhan, China.

**Corresponding author**

Guohua Yuan, email: [yuanguohua@whu.edu.cn](mailto:yuanguohua@whu.edu.cn)

Telephone: (+86)15871770625

^1^The State Key Laboratory Breeding Base of Basic Science of Stomatology and Key Laboratory for Oral Biomedicine of Ministry of Education, School and Hospital of Stomatology, Wuhan University, Luoyu Road, No. 237, Wuhan, China. 430079.

^2^Frontier Science Center for Immunology and Metabolism, Wuhan University, Donghu Road, No. 115, Wuhan, China. 430071.

**Supplemental Tables**

| **Genotype** | **Primers** | **Length of PCR products** |
| --- | --- | --- |
| **Wwp2 KO** | F: CGCTCCCAAACCTTAAAGGAG | Targeted: 225 bp |
|  | R: TACAAGCCATCTAGCACAGCA |  |
| **WT** | F: ACTGTTGATCTGGGAAGTGTG | WT: 352 bp |
|  | R: AACTAGAAGCACGTTTGTTAGAGG |  |

**Table S1.** The primers for genotyping of WT and Wwp2 KO mice.

| Genotype | Age | Experiments | Sample number  (n=) | Total number |
| --- | --- | --- | --- | --- |
| WT mice | PN1 | primary mDPCs isolation | 300 | 300 |
| WT mice | E13.5, E16.5, PN1, PN2, PN5, 2W | histological analysis | 3 for each age | 21 |
| Wwp2 KO mice | PN1 | primary mDPCs isolation for mRNA and protein analysis | 10 | 10 |
|  | PN5 | histological analysis | 8 | 8 |
|  | 2W | histological analysis, micro-CT, SEM | 8 | 8 |
|  | 8W | histological analysis, micro-CT | 8 | 8 |
| WT littermates  (control) | PN1 | primary mDPCs isolation for mRNA and protein analysis | 10 | 10 |
|  | PN5 | histological analysis | 8 | 8 |
|  | 2W | histological analysis, micro-CT, SEM | 8 | 8 |
|  | 8W | histological analysis, micro-CT | 8 | 8 |

**Table S2.** Animal samples details. The random allocation of mice to WT or Wwp2 KO mice was driven by Mendelian Inheritance.

| **Plasmid** | **Source** |
| --- | --- |
| pCMV6-Myc/DDK-Wwp2 | Origene |
| pCMV6-Myc/DDK-Wwp2C838A | self-constructed (1) |
| pCMV3-C-His-KLF5 | Sino Biological |
| pcDNA3.1-Pten | Miaoling |
| pGL3-Dspp-2.6 kb | Professor Shuo Chen (2) |
| pGL3-Dmp1-2.6 kb |  |
| pGL3-Basic | self-owned (1) |
| TK-renilla luciferase plasmid (pRL-TK) |  |

**Table S3.** The information of overexpression plasmids.

| **Gene** | **Primers** |
| --- | --- |
| Gapdh | F: 5’-TGTGTCCGTCGTGGATCTGA-3’ |
|  | R: 5’-TTGCTGTTGAAGTCGCAGGAG-3’ |
| Pten | F: 5’-AAGGGACGGACTGGTGTAATGATTTG-3’ |
|  | R: 5’-CGCCTCTGACTGGGAATTGTGAC-3’ |
| Wwp2 | F: 5’-GGAGATAGACATGAGCGACTGG-3’ |
|  | R: 5’-CAACAGGCAGACGGCAGGT-3’ |
| Klf5 | F: 5’-CGATTCACAACCCAAATTTACC-3’ |
|  | R: 5’-GTATGAGTCCTCAGGTGAGCTTTTA-3’ |
| Dspp | F: 5’-GTGGGATCATCAGCCAGTCAG-3’ |
|  | R: 5’-TGCCTTTGTTGGGACCTTCA-3’ |
| Dmp1 | F: 5’-ACCACAATACTGAATCTGAAAGCTC-3’ |
|  | R: 5’-TGCTGTCCGTGTGGTCACTA-3’ |
| Col1a1 | F: 5’-TGGCAAAGACGGACTCAAC-3’ |
|  | R: 5’-GGCAGGAAGCTGAAGTCATAA-3’ |

**Table S4.** The primers used for RT-PCR.

**Supplemental Figures**


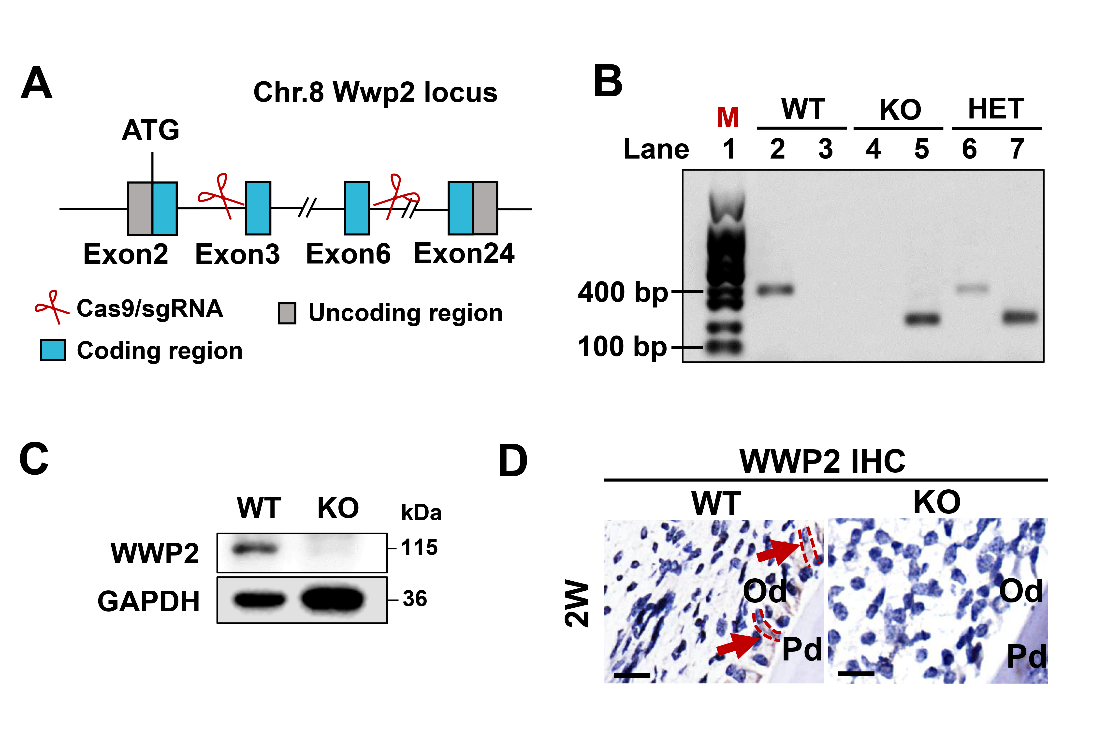


**Figure S1.** The establishment of Wwp2 KO mice.

**(A)** The knockout strategy of Wwp2 KO mice.

**(B)** The genotyping of Wwp2 KO mice.

**(C)** Ablation of WWP2 protein in Wwp2 KO mDPCs revealed by WB.

**(D)** IHC of WWP2 in WT and Wwp2 KO molars at 2W. Red arrows show the positive signals in odontoblasts of WT molars. Red dotted lines manifest the boundaries of odontoblasts.

Scale bar = 20 μm for (D). M, DNA marker; WT, wildtype; HET, heterozygote; Od, odontoblast; Pd, pre-dentin.


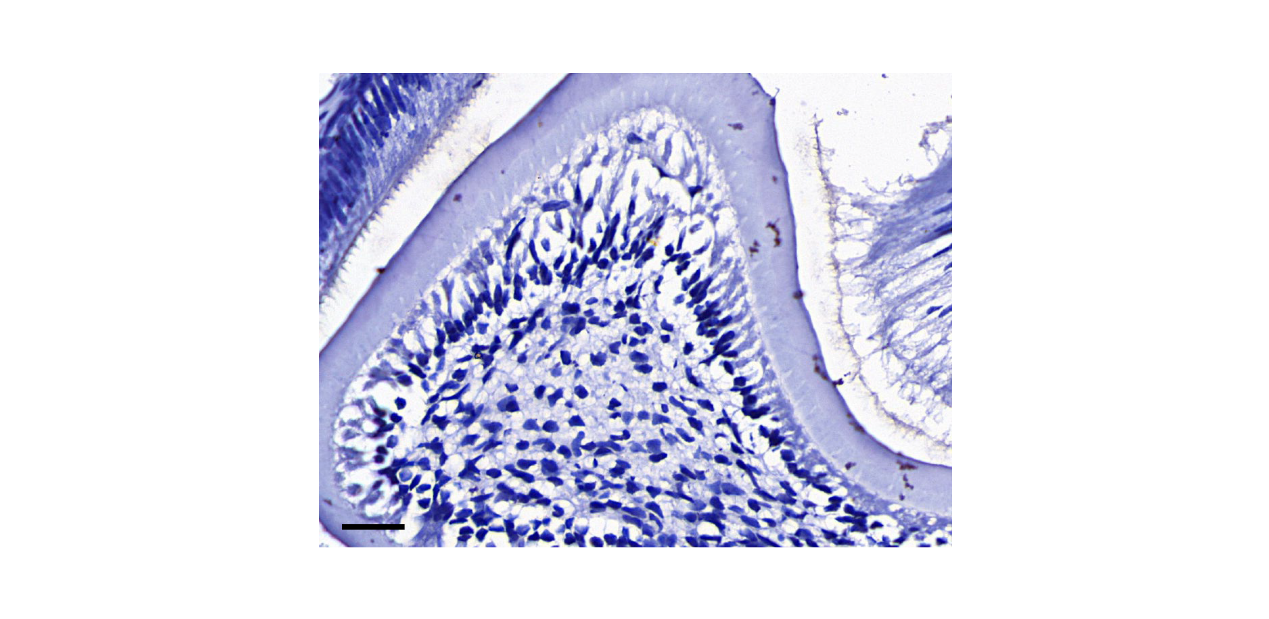


**Figure S2.** The negative control of IHC. The nonimmune control IgG was applied as the primary antibody. Scale bar = 50 μm.


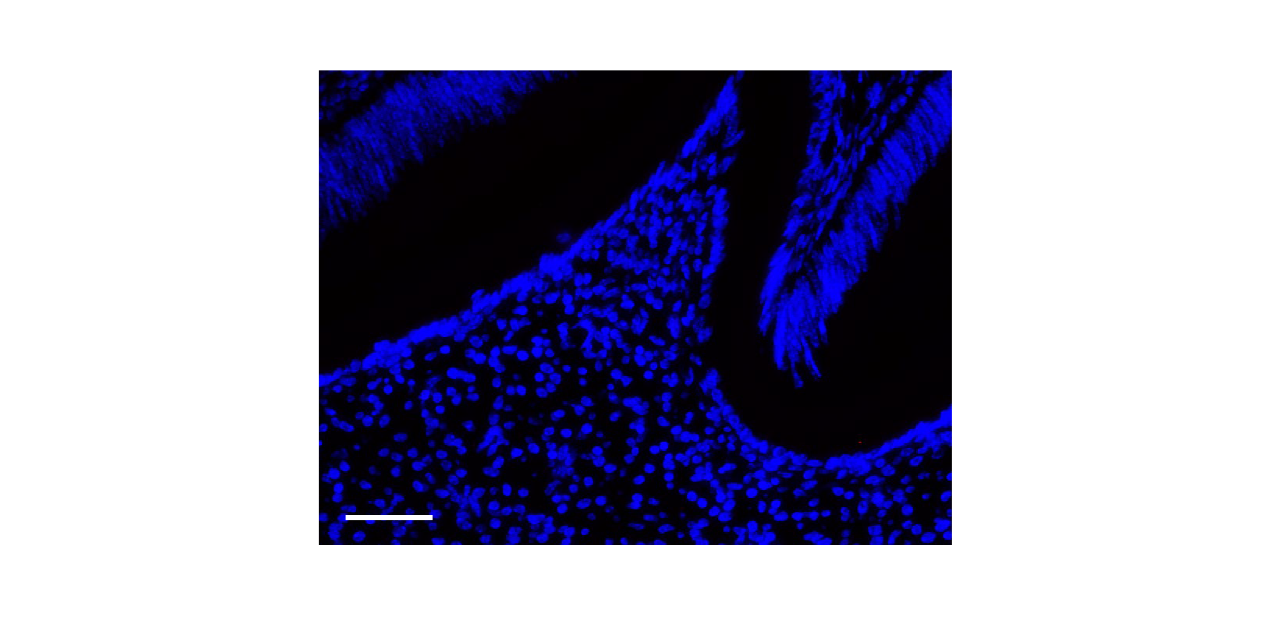


**Figure S3.** The negative control of IF. The nonimmune control IgG was applied as the primary antibody. Scale bar = 50 μm.


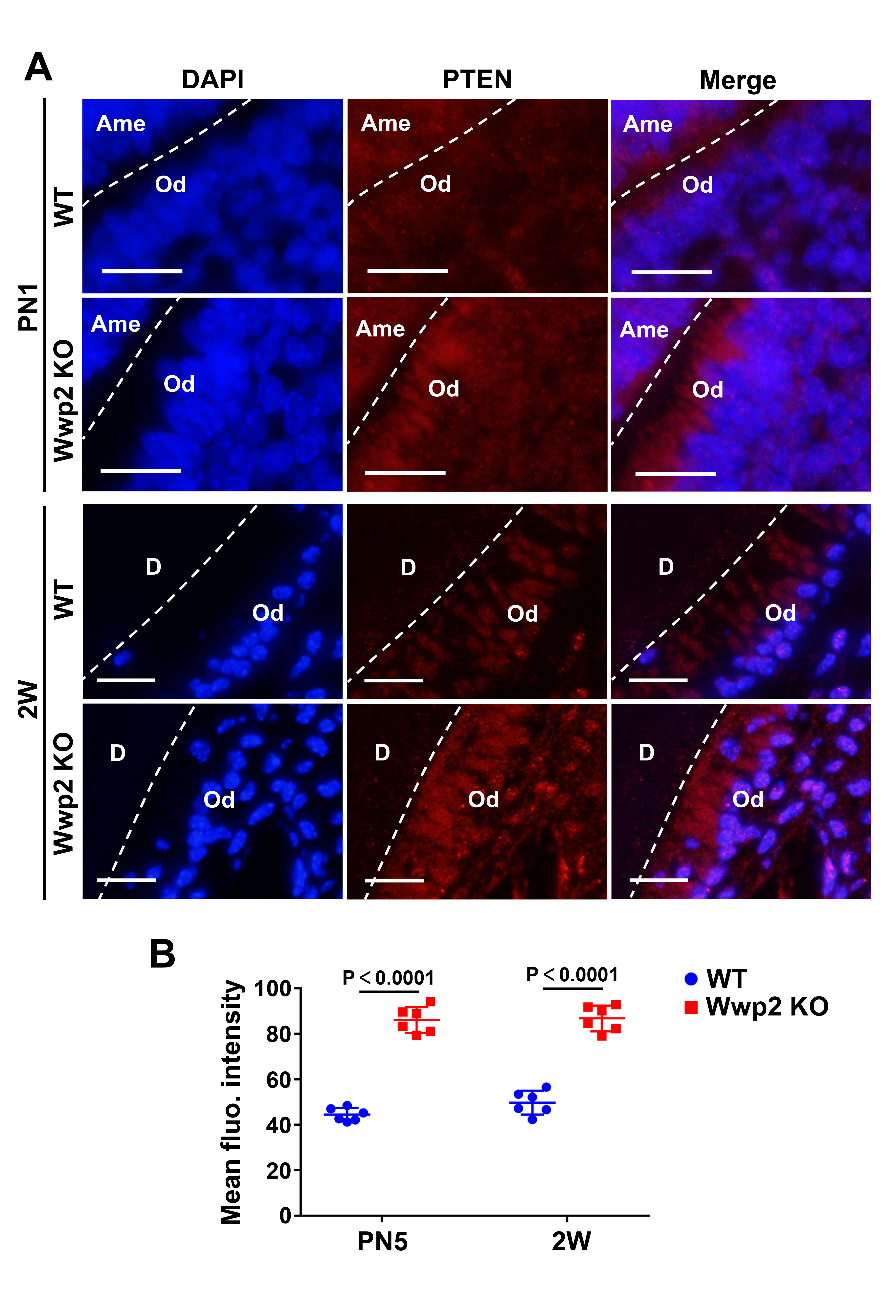


**Figure S4.** Increased PTEN expression in the odontoblast layer of Wwp2 KO mice.

**(A)** IF of PTEN in molars of wildtype and Wwp2 KO mice at PN1 and 2W.

**(B)** The mean fluorescence intensity of PTEN was quantified by Image J software in (B). n = 6; Student’s t test; Error bars represent SD.

Scale bar = 20 μm for (A). Ame, ameloblast; Od, odontoblast; D, dentin; fluo., fluorescence.


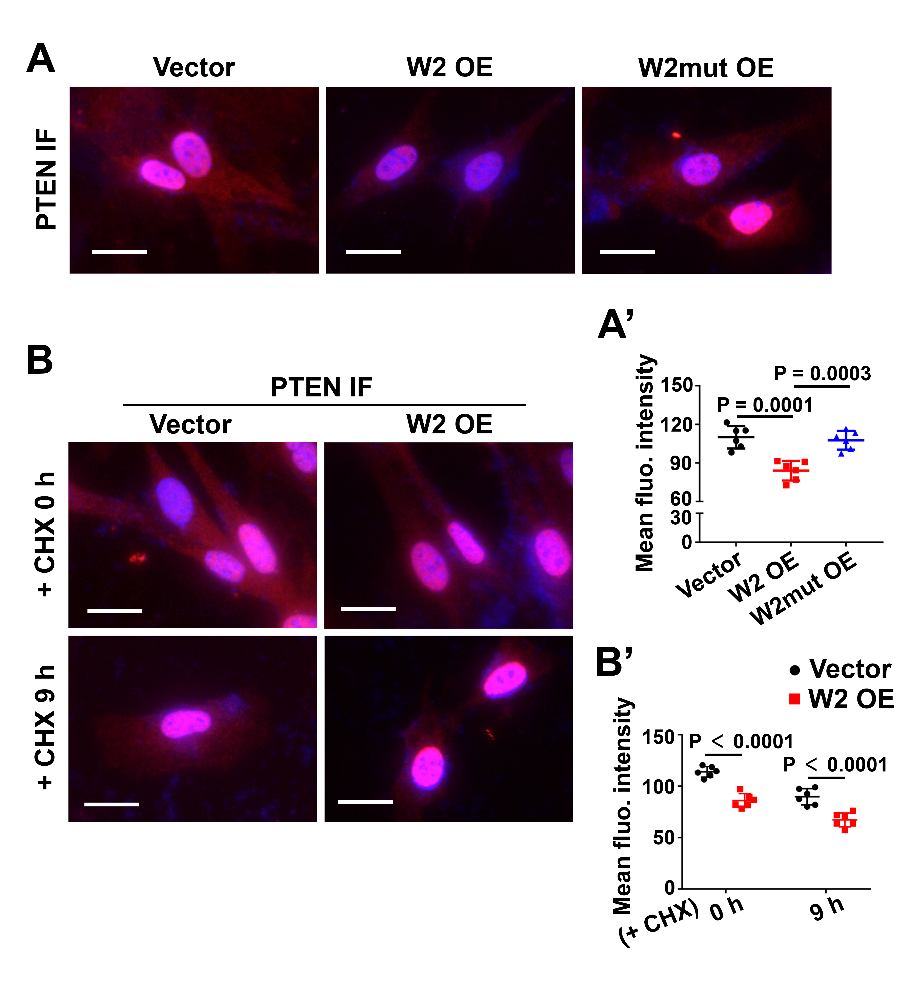


**Figure S5.** WWP2 promotes PTEN proteolysis dependent on its ligase-activity.

**(A and A’)** The protein level of PTEN was revealed by IF in vector-, Wwp2- and Wwp2 mutant-overexpressed mDPCs (A). The mean fluorescence intensity of PTEN was quantified by Image J software in (A’). n = 6; ANOVA; Error bars represent SD.

**(B and B’)** The protein level of PTEN was revealed by IF in vector- and Wwp2- overexpressed mDPCs. The cells were treated with CHX for 0 h or 9 h before being harvested (B). The mean fluorescence intensity of PTEN was quantified by Image J software in (B’). n = 6; Student’s t test; Error bars represent SD.

Scale bar = 20 μm for (A) and (B). W2, Wwp2; W2mut, Wwp2 ligase activity-dead mutant; OE, overexpression; fluo., fluorescence.


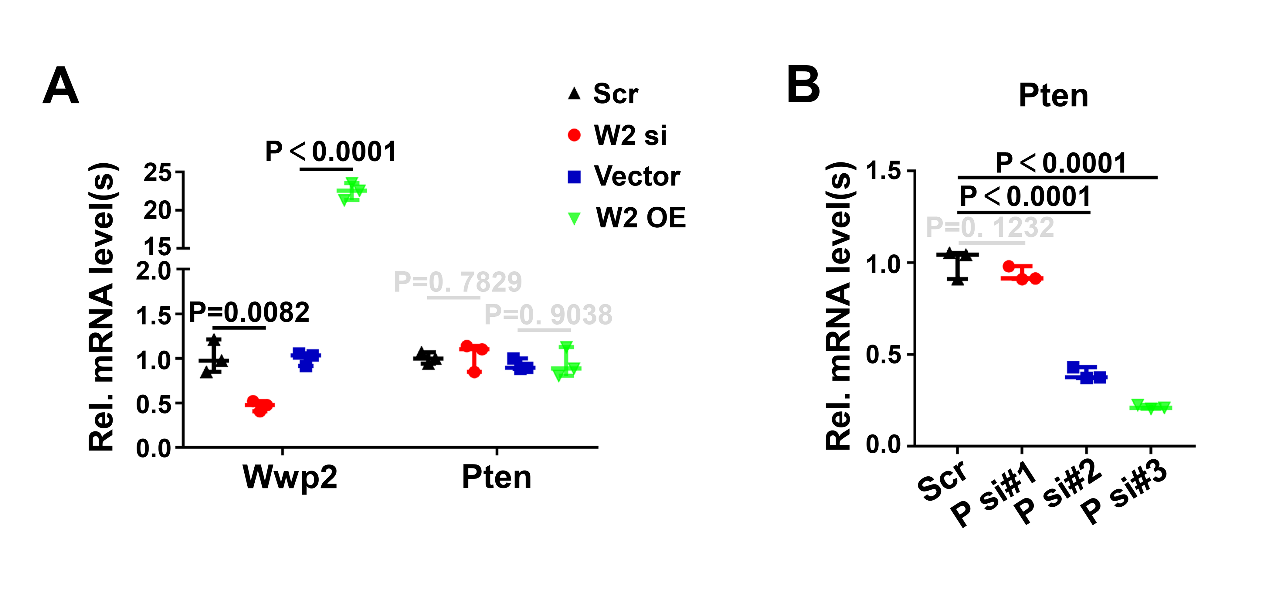


**Figure S6.** Neither knockdown or overexpression of Wwp2 changed the mRNA levels of Pten revealed by RT-PCR in (A) and the knockdown efficiency of Pten siRNAs was evaluated by RT-PCR in (B). n = 3; ANOVA; Error bars represent SD in both (A) and (B).

W2, Wwp2; Scr, scramble; Myc-W2, Myc-Wwp2; si, knockdown; OE, overexpression; Rel., relative.


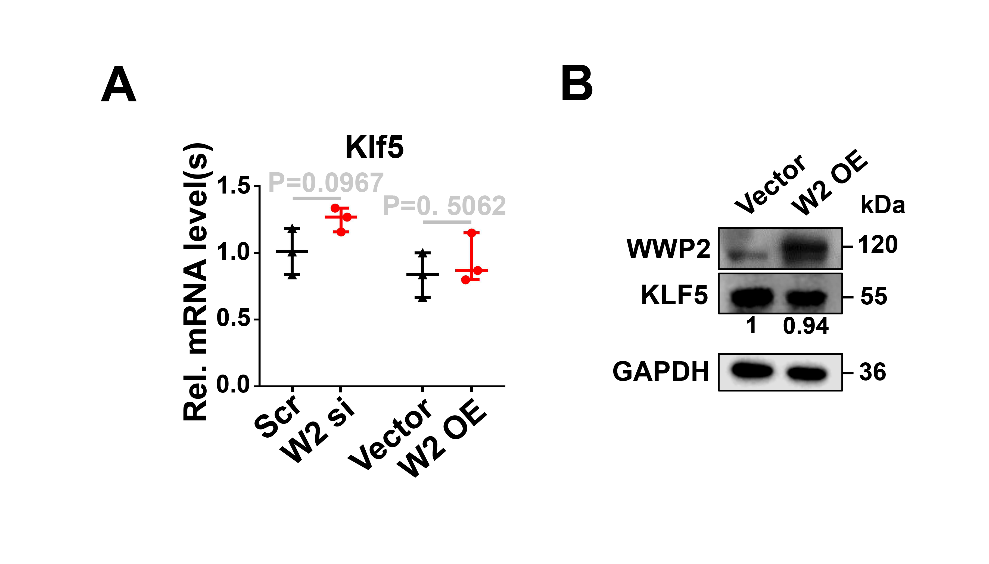


**Figure S7.** WWP2 showed no effect on the mRNA (A) and protein (B) levels of KLF5 revealed by RT-PCR and WB. n = 3; Student’s t test; Error bars represent SD in (A).

W2, Wwp2; Scr, scramble; si, knockdown; OE, overexpression; Rel., relative.

**References in Supporting Information**

1. Fu J, Zheng H, Xue Y, et al. WWP2 Promotes Odontoblastic Differentiation by Monoubiquitinating KLF5. *J Dent Res.* 2021;100(4):432-439.

2. Chen Z, Zhang Q, Wang H, et al. Klf5 Mediates Odontoblastic Differentiation through Regulating Dentin-Specific Extracellular Matrix Gene Expression during Mouse Tooth Development. *Sci Rep.* 2017;7:46746.
